# Protocol: evaluation of an optimised couples-focused intervention to increase testing for HIV in rural KwaZulu-Natal, South Africa, the Igugu Lethu (‘Our treasure’) cohort study

**DOI:** 10.1186/s12889-022-13894-3

**Published:** 2022-08-19

**Authors:** Nuala McGrath, Nathi Ngcobo, Zhixin Feng, Phillip Joseph, Pumla Dladla, Thulani Ngubane, Victoria Hosegood, Katherine Morton, Heidi Van Rooyen, Alastair Van Heerden

**Affiliations:** 1 CHERISH programme, School of Primary Care, Population Sciences and Medical Education, Faculty of Medicine, University of Southampton, Southampton, UK; 2 Department of Social Statistics & Demography, Faculty of Social Sciences, University of Southampton, Southampton, UK; 3 Clinical HIV Research Unit, University of the Witwatersrand, Johannesburg, South Africa; 4 School of Geography and Planning, Sun Yat-sen University, Guangzhou, China; 5 Human Sciences Research Council, Sweetwaters, Pietermaritzburg, KwaZulu-Natal South Africa; 6 School of Health Sciences, University of Southampton, Southampton, UK; 7 SAMRC-WITS Developmental Pathways for Health Research Unit, Faculty of Health Sciences, University of the Witwatersrand, Johannesburg, South Africa

**Keywords:** Couples HIV testing and counselling, Couples-focused intervention, Behavioural intervention, Couples health, South Africa

## Abstract

**Background:**

Between 2012 and 2015, the Uthando Lwethu (UL) study demonstrated that a theory-based behavioural couples-focused intervention significantly increased participation in couples HIV testing and counselling (CHTC) among South African couples who had never previously tested for HIV together or mutually disclosed their HIV status, 42% compared to 12% of the control group at 9 months follow-up. Although effective, we were nonetheless concerned that in this high prevalence setting the majority (58%) of intervention couples chose not to test together. In response we optimised the UL intervention and in a new study, ‘Igugu Lethu’, we are evaluating the success of the optimised intervention in promoting CHTC.

**Methods:**

One hundred eighty heterosexual couples, who have been in a relationship together for at least 6 months, are being recruited and offered the optimised couples-focused intervention. In the Igugu Lethu study, we have expanded the health screening visit offered to couples to include other health conditions in addition to CHTC. Enrolled couples who choose to schedule CHTC will also have the opportunity to undertake a random blood glucose test, blood pressure and BMI measurements, and self-sample for STI testing as part of their health screening. Individual surveys are administered at baseline, 4 weeks and 4 months follow-up. The proportion of couples who decide to test together for HIV will be compared to the results of the intervention arm in the UL study (historical controls). To facilitate this comparison, we will use the same recruitment and follow-up strategies in the same community as the previous UL study.

**Discussion:**

By strengthening communication and functioning within the relationship, the Igugu Lethu study, like the previous UL study, aims to transform the motivation of individual partners from a focus on their own health to shared health as a couple. The Igugu Lethu study findings will answer whether the optimised couples-focused behavioural intervention and offering CHTC as part of a broader health screening for couples can increase uptake of CHTC by 40%, an outcome that would be highly desirable in populations with high HIV prevalence.

**Trial registration:**

Retrospectively registered. ISRCTN Registry ISRCTN 46162564 Registered on 26th May 2022.

**Supplementary Information:**

The online version contains supplementary material available at 10.1186/s12889-022-13894-3.

## Background

South Africa has experienced one of the world’s most severe generalised HIV epidemics. In 2011, HIV prevalence was 29% among resident adults aged 15–49 years in KwaZulu-Natal (KZN) [1]. Between 2004 and 2011 HIV incidence in KZN was 2.63 per 100 person-years (95% CI 2.50 to 2.77) [2], with the majority of new infections occurring within heterosexual partnerships. Current recommendations are that HIV treatment should be started as soon as diagnosis is made [3]. Therefore, regular HIV testing remains crucial to achieving high levels of treatment coverage and the gateway for treatment and prevention pathways (e.g. pre-exposure prophylaxis). South African HIV testing campaigns have increased individual knowledge of HIV status but have not overcome barriers to repeat HIV testing and disclosure of HIV status to sexual partners [4]. Gender-specific concerns about HIV testing and treatment are well documented [5]. Thus, it is important that universal test and treat and treatment as prevention efforts are sensitive to men and women’s relationships with partners and family. In couples HIV testing and counselling (CHTC), couples are counselled, tested and receive their HIV results together [6]. In South Africa, as elsewhere in sub-Saharan Africa, the provision and uptake of CHTC remains low [7]. CHTC accomplishes two important goals. First, knowledge of each other’s HIV status can facilitate risk-reduction behaviour within partnerships via effecting positive changes (e.g., condom use) in sexual behaviour with primary and any concurrent partners. Second, knowledge of HIV status can increase access to treatment and care for HIV-positive individuals, as well as reinforce behavioural choices (e.g., limiting concurrent partners) to stay HIV-negative.

Between 2012 and 2015, the Uthando Lwethu Study tested the efficacy of an interdependence theory-based behavioural couples-focused intervention to increase participation in CHTC in South Africa among couples who had never tested for HIV together or mutually disclosed their HIV status [8]. Three hundred thirty-two couples were randomised to the intervention arm (168 couples) or the control arm (164 couples). The intervention arm received two group sessions and four couples counselling sessions, focused on problem-solving and communication skills and was highly effective in increasing the proportion of couples that decided to test for HIV together within 9 months after enrolment, (42% v. 12% [*p* < 0.001]) compared to the control group [9]. Concerned that, in a high HIV prevalence and continued HIV incidence setting [10], 58% of intervention couples did not choose to test together during the Uthando Lwethu study, we optimised the Uthando Lwethu intervention using the Person-Based Approach to intervention development and optimisation [11]. The details of the process of optimising the intervention have been published elsewhere [12]. In brief, a qualitative study was conducted with 20 purposively sampled couples who were formerly enrolled in the intervention arm of the Uthando Lwethu Study, and with 5 study staff who delivered intervention components, with the aim of exploring experiences of the intervention and barriers to testing. The past participants sampled included couples who did not attend couples counselling sessions and did not test for HIV together, some couples who attended all couples counselling sessions and tested for HIV together during follow-up, and others who only took up part of the intervention, with or without deciding to test for HIV together. Developing guiding principles and a logic model that showed how the optimised intervention would increase uptake to CHTC ensured that optimisations were grounded in the local context and drew on theoretical constructs [12]. The optimised intervention still involves two group sessions and up to four couples’ counselling sessions but differs from the Uthando Lwethu intervention in session content, involving community members speaking publicly about their HIV testing history and HIV status, and the possibility that the number of counselling sessions provided will be less if the couple engages in couples HIV testing at an earlier point in the intervention.

In South Africa, rates of intimate partner violence (IPV) reported in the literature are high [13]. The most recent Demographic Health Survey in South Africa (2016) reports one in four (26%) ever-partnered women age 18 or older have experienced physical, sexual, or emotional violence committed by a partner in their lifetime [14]. In the Uthando Lwethu study, among the 448 couples screened for eligibility, there were almost no reports of a history of IPV, and no reports of IPV during study follow-up were recorded. The Uthando Lwethu study excluded couples with a recent history of IPV (within last 6 months) because it was recognised that such couples would need both a different intervention and, support by staff with specific training and experience of counselling couples experiencing ongoing IPV. However, the Uthando Lwethu study included participants where an experience of IPV had occurred less recently. Thus, minimising selective exclusion of people at a high risk of HIV-infection.

To normalise HIV testing in research studies, in recent years HIV testing is increasingly offered as part of a broader health check [15–17], which led us to include screening for other conditions as part of our CHTC offer during follow-up, and to promote this as an opportunity for ‘couples health screening’.

### Study setting

**Fig. 1:**
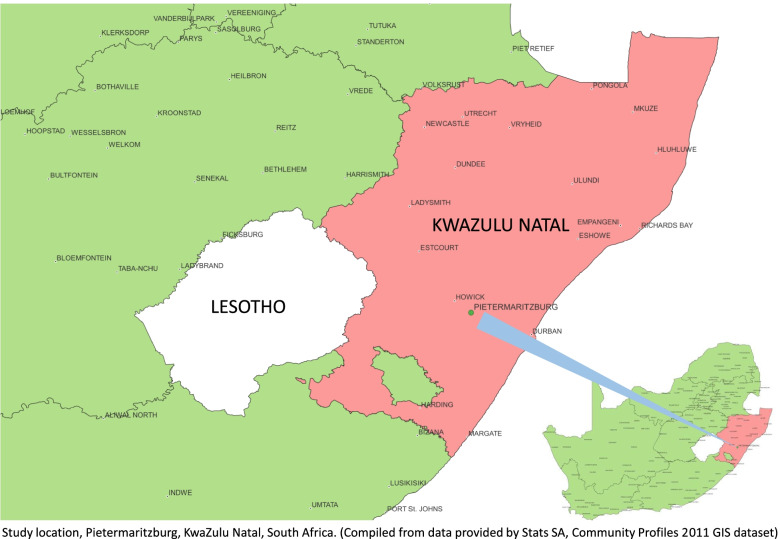
Study location, Pietermaritzburg, KwaZulu-Natal, South Africa. Compiled from data provided by Stats SA, Community Profiles 2011 dataset [18]

As was the Uthando Lwethu study [8, 9], the Igugu Lethu study will be conducted at Human Sciences Research Council’s (HSRC) Sweetwaters research site in the rural Sweetwaters community west of the capital of KwaZulu-Natal, Pietermaritzburg (Fig. 1, created by P. Joseph [18]). Part of the Greater Edendale Area, the community of around 600,000 people, is representative of the many Zulu communities in the province. The Sweetwaters community experiences high rates of infectious diseases such as HIV and TB, as well as non-communicable disease [19, 20]. The only healthcare options available to most Sweetwaters community members are free district clinics run by the provincial Department of Health and dispersed mobile clinic providers operated by NGOs; private health clinics are not an option for the majority of community members, given the low employment rate and low median monthly household income.

In terms of non-communicable diseases (NCDs), adult diabetes is now a major problem in some countries in sub-Saharan Africa [21]. In the next decade adult diabetes is anticipated to become one of the most challenging public health conditions throughout the region, partly due to effective HIV treatment extending life expectancy, as well as changes in diets and lifestyles [22]. The age-standardised prevalence of diabetes in South Africans aged 15+ was 10.1% in 2011, 90–95% of which is type 2 diabetes [23]. The high prevalence of diabetes complications and mortality in adults with diabetes typically results from poor glycaemic control [22]. In South Africa, diabetes was the second largest underlying cause of death among adults aged 15–44 years in 2015 [24]. South Africa’s Strategic Plan for the Prevention and Control of Non-Communicable Diseases 2013–17 strategy [25] set a target of a 30% increase in the percentage of diabetic patients who are well controlled by 2020.

Obesity and hypertension are recognised as leading risk factors for cardiac diseases in low- and middle-income countries. In rural KwaZulu-Natal (KZN), population-based studies have reported the measured prevalence of obesity (BMI > 30 kg/m2) and hypertension (systolic or diastolic blood pressure > 140 or 90 mmHg, respectively) at 32 and 24% respectively among adults aged 15–50 years in 2004 [26]. Other population studies in KZN have documented similar levels of obesity and hypertension and documented pronounced gender differences, with 6.5 times higher prevalence of obesity in women and 1.4 times higher prevalence of hypertension in women than in men [19, 27]. The dual epidemics of communicable and non-communicable diseases in sub-Saharan Africa mean that many adults are living with more than one diagnosis, often managing co-morbidities alongside HIV. In a study of adults attending a primary care clinic in Khayelitsha, Western Cape, South Africa, in 2013 with at least one chronic illness (HIV, tuberculosis, diabetes, and hypertension), hypertension was the most common morbidity (65%), and 22.6% of patients had multimorbidity, with an increasing prevalence with age [28].

The four curable sexually transmitted infections (STIs, chlamydia, gonorrhoea, syphilis, and trichomoniasis) are prevalent in KZN, with significant sexual and reproductive health consequences including genital symptoms, pregnancy complications, infertility, and enhanced HIV transmission [29]. The standard of care for STIs in sub-Saharan Africa is syndromic management i.e. presumptive treatment without confirmatory laboratory tests [30]. The importance of partner notification and treatment is emphasised in STI clinical care but the strategy is passive. Offering lab STI-testing to couples provides opportunities to treat asymptomatic cases, facilitate partner treatment and, promote reproductive health, and fits well with the aims of South Africa’s National Strategic Plan on HIV, TB and STIs (2017–2022) [31].

At the Sweetwaters site, it is standard protocol to register all individuals who interact with a research study by fingerprint scanning to avoid multiple screening or enrolment of the same person. This biometric approach has been successfully implemented in several studies [17, 32, 33].

## Methods / design

The current protocol is version 1.3 dated 3rd November 2020. All protocol modifications will be communicated to the Human Sciences Research Council and the University of Southampton Ethics Committees for approval before they are implemented.

### Study objectives

The primary objective of the Igugu Lethu study is to measure the efficacy of our optimised theory-based and culturally appropriate couples-focused intervention on the uptake of HIV testing as a couple. We also have four secondary objectives. First, we aim to investigate the extent to which relationship dynamics are factors in achieving the outcome of couples’ uptake of HIV testing together. Second, we aim to explore whether couples take up other testing opportunities offered during the health screening visit, and the frequency of couples’ health concordance (the same status) for the different tests conducted. Third, we aim to identify operational reasons for failure or success in implementing the intervention and of the study as a whole. Finally, we aim to explore the penile microbiome functional characteristics of male partners and the vaginal microbiome functional characteristics of female partners and concordance in these characteristics within couples.

### Study design

**Fig. 2:**
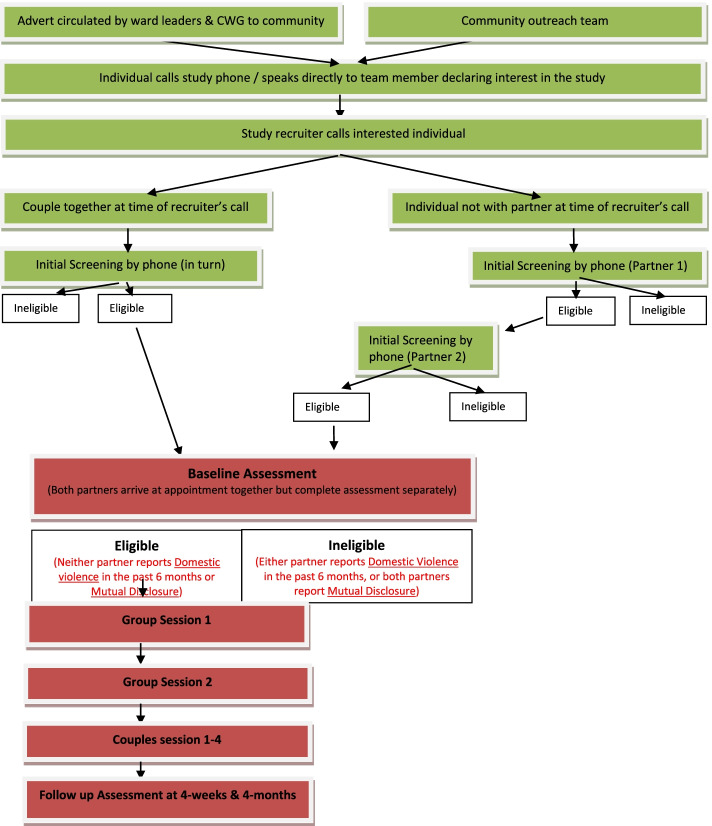
Study flow

Igugu Lethu is a prospective cohort study. Figure 2 summarises the study flow, including two stages of recruitment (initial screening and baseline screening) to determine the eligibility of couples to participate in the Igugu Lethu study, and the intervention sessions and follow-up assessments for those enrolled.

To maintain comparability with the Uthando Lwethu study, we will use the same eligibility criteria and similar recruitment and follow-up strategies used in the Uthando Lwethu study, with some adjustments for SARS-CoV-2 risk mitigation.

### Eligibility criteria

#### Inclusion criteria

Couples are considered eligible for baseline screening if they meet these inclusion criteria:

1. Both partners 18 years and older
2. In a primary relationship with an opposite sex partner. [Defined as: “Are you currently in a relationship with a person of the opposite sex to whom you feel committed above anyone else and with whom you have had sexual relations”]
3. Both partners report the relationship length has lasted at least 6 months
4. Both partners are willing and able to provide informed consent

#### Exclusion criteria

Couples are considered ineligible for baseline screening if they meet these exclusion criteria:

1. Both partners report participating in couples-focused testing for HIV with their current partner [Defined as: Both partners tested for HIV at the same time and disclosed the results].
2. Either partner reports their current marital status as polygamous.
3. Either partner reports participating in the Uthando Lwethu study
4. Either partner fails to answer exploratory relationship-focused questions about their partner.

As in the Uthando Lwethu study, couples who complete baseline screening will be considered ineligible to participate if either of the following additional exclusion criteria is determined to have been met:

1. Either partner reports intimate partner violence (as actor or recipient) within the past 6 months with their current partner
2. Both partners have ever disclosed HIV test results to each other [via individual or couples testing].

### Recruitment

#### Initial screening

To identify potential participants for initial screening, ward leaders, community working group members, and other contacts in the target community will circulate our study advert using WhatsApp, text etc. Interested potential participants are asked to contact the study team via the contact details included in the advert. The study will also be promoted by including the study advert in the local newspapers and on local radio, and by the HSRC community outreach team as they attend meetings and events in the community and in public areas in the community, such as markets, churches, workplaces, bus and taxi stands, and community centres using a loudhailer. If a couple or an individual is interested in participating in the study, recruiters will determine their eligibility through an initial screening survey over the phone. Recruiters will emphasise that both partners must complete the initial screening to determine if the couple is eligible for the baseline screening.

#### Baseline screening

Once both partners have completed the initial screening and if they are both eligible, a staff member will contact them to schedule a baseline screening visit. Experience during the Uthando Lwethu study showed that some couples who attended a screening visit were pretending to be couples in order to obtain the reimbursement. To discourage pretend couples, at the beginning of the baseline screening appointment, the couple will be individually asked questions of each partner to check if each partner knows the other (without recording the answers). The baseline screening continues only for couples where answers given by both partners are consistent.

Once couples have given informed consent, each partner will be asked to register with fingerprint scanning, and then a baseline survey will be administered, which takes approximately 60 min to complete. If either partner’s score suggests severe violence has occurred within the relationship in the last 6 months or both partners report HIV disclosure to each other, the couple will be ineligible to continue the study. If neither partner reports severe domestic violence in the relationship, and a maximum of one partner reports HIV disclosure, the couple is eligible to continue the Igugu Lethu study.

### Intervention

The proposed optimised behavioural intervention consists of up to six sessions (one group session in which couples are initially together and subsequently split into single-gender subgroups, a second single-gender group session, and four couples’ counselling sessions). The intervention is hypothesised to provide information and education around HIV testing and treatment, transform couples’ motivation for looking after their health as a couple rather than as individuals, as well as to improve communication, intimacy and trust necessary for mutual decision-making about behaviours related to sexual risk behaviour and testing for HIV.

Couples will not be reimbursed for the intervention sessions as these are perceived to offer a benefit to couples in and of themselves, and this offers a more feasible set-up to scale-up and implement more widely. This is different to the Uthando Lwethu study which offered reimbursement of ZAR50 (approximately GBP 3.00, as of January 2014) per person per session for all intervention sessions.

#### Group session 1

This first group session will be comprised of a half-day workshop focusing on general health issues in couples, including health conditions assessed at the general couples health screening (blood pressure, diabetes, curable STIs, obesity and HIV). Both partners participate together in the first section of the workshop, with approximately 15–20 couples in each group. Subsequently, the couples will be split into male-only and female-only sub-groups to begin discussing sexual health, family planning, HIV awareness, and benefits and barriers to testing. Community members living with HIV and others who have recently tested HIV negative, and have previously spoken publicly about their HIV testing history and HIV status, will be invited to attend and facilitate a dialogue around couples testing and impact on relationships during the single-gender session.

#### Group session 2

Approximately 1 week after completion of the first half-day workshop, couples will receive a second half-day workshop on couples dynamics, communication skills, and reproductive health issues (e.g., sexual health, HIV). The second workshop will be organised as separate parallel men- and women-only group sessions to facilitate discussion. The groups will be run by a gender-matched facilitator. In the single-sex group session, participants will undertake exercises on topics including couples’ dynamics, such as power, trust, commitment, gender norms and communication skills training. Refreshments will be provided for study participants.

#### Couples Counselling sessions

Couples will receive up to four couples’ counselling sessions, each lasting 1 to 2 h. These sessions will be led by a trained counsellor. The sessions include communication skills training, problem-solving, setting health-related relationship goals, and exploring perceived benefits and barriers to HIV testing within the couple. Each session will be spaced approximately 1 week apart at times convenient for couples.

If the couple engages in couples HIV testing at an earlier point in the intervention, the counselling structure will change, such that if a couple tests together after 1–2 counselling sessions, they will be offered one final counselling session, which would include the content of counselling session 4 in terms of planning for their future together.

If a couple misses a session, staff will call each partner to reschedule the appointment. If the couple cannot be reached, and they have consented to being visited at their home, a staff member will go to their home to follow up. If a couple has not completed the intervention sessions within 3-months of group session 1, no further intervention sessions will be arranged for this couple. Based on our experience in the Uthando Lwethu study, if a couple is motivated to take part in the intervention, all sessions will be completed within this 3-month window.

##### Couples session one

In the first couples counselling session, couples will re-visit and practice the communication technique introduced in the second group session and describe to each other what happened in their single-gender sessions. Couples will explore the basic expectations they have in their relationship related to their health and set health-related relationship goals and plans for how they will meet these. In addition, couples will engage in exercises to learn and practice helpful problem-solving strategies. The couples will also engage in a commitment exercise to remind them of their partner’s and their relationship’s positive aspects.

##### Couples session two

Couples will begin the second couples counselling session with a brief check-in about their time since the last session and ask about progress in their health-related relationship goal. The counsellor will also help the couple review the first counselling session.

The counsellor will help the couple discuss the barriers or facilitators they face in meeting their expectations around HIV, risk, and couples testing. Couples will identify a barrier they would like to address during the session. The couples will then use the communication technique to engage in problem-solving exercises to talk about the ir identified barrier.

##### Couples session three

In the third couples counselling session, the counsellor will check in with couples about their time since the last session and ask about progress in their health-related relationship goal. When the couple has finished this review, if appropriate, they will be asked to name two other barriers they face in meeting each other’s expectations related to HIV. For the remainder of the session, the couples will use the communication technique to engage in problem-solving exercises to address their identified barriers.

##### Couples session four

The fourth and final couples counselling session will begin with a brief check-in with the couple about their time since the previous counselling session, ask about progress in their health-related relationship goal and their thoughts on what they have learned over the course of the intervention.

The counsellor will facilitate a discussion about the couple’s plan to move forward and use the skills they learned in their counselling sessions.

The couple will also discuss upcoming issues and events that may present a challenge for them and how they can remember to use the skills they gained during the intervention. Specifically, the counsellor will ask the couple to talk through the skills they have learned and resources they identified such as support from family or community members.

Finally, the counsellor will wrap up the session by summarising the specific issues that the couple worked on during the counselling sessions and the tools they used to address them. The counsellor will help the couple set goals for the future in light of the new tools and resources they gained and identified.

### Study procedures and follow-up

#### Baseline survey

The baseline survey includes measures of domestic violence and mutual disclosure to confirm the couple’s eligibility. In addition, the survey includes measures of relationship domains such as satisfaction, communication and measures about HIV and reproductive health (e.g., fertility intentions, HIV knowledge and risk perception, and sexual behaviour). All measures were previously used in Uthando Lwethu. We have dropped some demographic questions from the Uthando Lwethu baseline questionnaire, which were deemed unnecessary, and added questions on self-reported health status (e.g. raised blood pressure and diabetes) and health behaviours, as recommended by the WHO STEPs protocol [34].

#### Enrolment in the intervention

Couples will arrive together at the venue for the first group session. In the Uthando Lwethu study approximately two-thirds of couples who remained eligible after the baseline survey joined a group 1 session. Thus, our target of enrolling 180 couples refers to the number of couples attending group session 1.

#### Follow- up

All enrolled couples will have follow-up assessments in person or by phone at 4-weeks and at 4-months after the Group 1 session. The follow-up questionnaires used at 4-weeks and 4-months are similar to the follow-up questionnaire used in the Uthando Lwethu study. Each follow-up visit will follow similar procedures as detailed for the baseline survey. Each member of the couple will be interviewed separately, ideally at the same time, by a gender-matched interviewer.

During the baseline consent process, participants will be asked if they are willing to be contacted by phone for appointment reminders, as well as follow ups for missed appointments. They will also be asked if they are willing to be visited at home if they miss a study visit. After participants have consented to participate in the study, they will be asked to fill out a tracking form. This form will collect contact details, to facilitate reminder calls for scheduled appointments, as well as follow up calls for missed appointments. Additionally, if the participant agreed to be visited at home after missing a study visit, they will provide detailed information about the location of their primary residence, as well as any alternative residences. Contact information will be updated during each follow up visit.

At the end of the 4-month interview, participants will receive a certificate of study completion, an end of study information sheet and they will be asked to provide written consent to contact for future studies.

#### Reimbursement

Couples will be reimbursed ZAR80 (approximately GBP 4.00, as of January 2021) for each data collection appointment (baseline questionnaire, 4-weeks follow-up and 4-month follow-up), mirroring what was done in the Uthando Lwethu study. Reimbursement will be by universal cell-phone voucher sent electronically. Transport will be provided to all intervention sessions and data collection visits, so there will be no transport costs incurred by participants.

#### Couples health screening visit

Couples enrolled in the Igugu Lethu study may schedule an appointment for a couples’ health screening visit at any time after the first group session, once they feel ready to participate in HIV testing and counselling together. The health screening visit will only proceed if, on the appointment date, both partners consent to HIV testing together. All other tests offered are optional. The visit will be conducted to avoid days when the female partner is menstruating. If couples report testing for HIV together as a couple at an alternate venue during the Igugu Lethu study, the couple will be encouraged to undergo the couples health screening visit before completion of study participation.

Informed consent will be taken for each of the health screening procedures (HIV test, random blood glucose (RBG) tests, blood pressure and body mass index (BMI) measurements, and self-sampling for sexually transmitted infection testing) at the start of the visit from each couple by the study counsellor.

At the couples health screening visit, height and weight will be measured, and BMI will be calculated for each partner using an electronic scale (Scale Digital BMI 1110H SOAMAA). Blood pressure will be measured by a study counsellor using Brand Axcess BP 1359 devices while the participant is seated, and three consecutive readings will be averaged [35], in line with recommendations by the American Heart Association.

The counsellor will explain the need for self-collected samples to test for chlamydia, gonorrhoea, and trichomoniasis. The counsellor will explain and demonstrate the procedure for obtaining the self-collected vaginal swabs to the female member of the couple and will ask her to self-collect two sequential vaginal swabs behind a medical curtain for privacy. A nurse will be on call if assistance is needed. For men, the counsellor will explain and demonstrate how to obtain a self-collected penile swab, rotating a flocked swab twice around the full circumference of the penis at the *coronal sulcus*, the junction between the glans and the shaft of the penis. A urine sample will be requested for testing *C. trachomatis, N. gonorrhoeae*, and *T. vaginalis* infections. After the sample collection, samples will be transported to a commercial accredited laboratory in cool boxes. The couples health screening questionnaire includes questions on the acceptability of self-sampling.

The counsellor will conduct pre-test couples counselling for HIV prior to taking blood samples from each partner. A push button lancet safety needle will be used to draw blood from the finger for simultaneous HIV testing, random plasma glucose (RPG) using One Call Plus (ref G113–214) test strips: one Call Plus 50: G133–111 LOT 1690533, and a dry blood spot card will be made to conduct an initial screener test for syphilis (*Treponema pallidum* particle agglutination assay (TPPA test). For HIV testing, the nurse will follow a serial testing algorithm for rapid HIV testing according to South African national guidelines. All participants will be first tested with an advanced one-step anti-HIV test, and specimens that are non-reactive will be considered HIV-negative. Any specimens that are reactive on the first test shall be tested again using a diagnostic kit for HIV antibody (colloidal gold) v2 test. Where specimens are reactive on both the first and the second assays, the results will be reported as HIV-positive. Specimens that are reactive on the first assay but non-reactive on the second assay will receive an ELISA laboratory test and be recorded as discordant. Once HIV test results are available for both partners, the couple will receive post-HIV test counselling with the counsellor. Active referral (i.e. the study staff will make an appointment or accompany the participant to an appointment) will be made for HIV.

The counsellor will give all other results with appropriate interpretation, counselling messages and Department of Health information pamphlets to the couple. The NCD counselling messages will be based on WHO Guidelines for Primary Care in Low-Resource Settings [36], and Section 4.1 of the Standard Treatment Guidelines and Essential Medicines List for South Africa-Primary Health Care Level 2018 Edition [37]. The counsellor will use the BMI-based CVD risk Framingham Heart Study tool to calculate for participants their 10-year risk of a fatal or non-fatal cardiovascular event (myocardial infarction or stroke), according to age, sex, blood pressure, presence or absence of diabetes, and smoking status, and will use this score when counselling about lifestyle changes [38]. In addition to lifestyle counselling, active referral to the clinic will be made for individuals who have systolic blood pressure (SBP) ≥ 180 mmHg or diastolic blood pressure (DBP) ≥ 110 mmHg, and a letter of referral (passive referral) will be given to those with moderate hypertension (SBP: 160–179 mmHg, DBP 100–109 mmHg), suggesting they have a repeat blood measure in 2 weeks. For individuals with an RBG result of > 11.0 mmol/L, we will ask questions to determine if they have symptoms indicating diabetes. If their answers indicate symptoms, they will be actively referred to a clinic. If they do not report symptoms, they will be given a letter of referral (passive referral) requesting that the clinic tests their fasting blood glucose level. Local clinics have agreed to receive such referred cases.

A date for the return (STI results) visit will be scheduled before the couple leave the health screening visit. The preferred mode of contact (e.g. telephone call, text or WhatsApp Messenger or in persons) and ideal hours for contact will be recorded for each couple, so that they can be notified whether they need to physically attend their STI results appointment when their STI results and those of their partner become available. Couples will be informed that if we find a curable STI and three attempts to contact them by their preferred mode of contact fail, a study staff member will attempt to find them. Couples who are referred to clinic during the couples’ health screening visit because one or more test results are ‘out of range’, will be asked to return to the appointment in 2 weeks regardless of positive or negative STI results.

#### STI results visit

For all couples who have at least one ‘out of range’ test during the health screening visit, the STI results visit provides an opportunity for the study team to document whether the couple has acted on any referral made, and if not, to counsel them again regarding the benefits of linking to care. Couples will also receive their STI results together at this visit.

The South African STI Management Guidelines (2015) [39] only provide guidance for syndromic management of STIs i.e. treatment based on presenting STI symptoms, with the exception of syphilis treatment guidelines based on laboratory testing. Therefore, we will use the British Association for Sexual Health and HIV guidelines for the treatment of laboratory diagnosed (i.e. asymptomatic) *N. gonorrhoeae, C. trachomatis* and *T. vaginalis* infections by a nurse, which we have used in a recent STI prevalence study in the same province of South Africa [29].

Samples showing a reactive treponemal test may represent past/treated syphilis or active syphilis. Thus, all positive results from the syphilis screener test on DBS will be referred to the PHC for venous blood testing for syphilis.

#### Laboratory procedures

Samples will be transported on the same day to the accredited laboratory. The dried blood spot will be tested for syphilis using a *Treponema pallidum* particle agglutination assay (TPPA test) [40]. Urine from men and one vaginal swab from each woman will be used for testing of *C. trachomatis, N. gonorrhoeae*, *and T. vaginalis* with DNA amplification methods. The remaining extracted DNA from these samples, along with the self-collected penile swab from male partners and the second vaginal swab from female partners, will be stored at − 80 degrees for later microbiome analysis.

After re-suspending and extracting DNA from stored penile and vaginal swabs, a fluorescent dye (Invitrogen QuantIT high sensitivity dsDNA) will be used to calculate DNA mass/volume which will then be extrapolated to mass/swab. A real-time (quantitative) PCR of the bacterial 16S ribosomal RNA gene will be used to estimate the number of bacterial genomes with linear dilutions of a standard.

After re-suspending and centrifuging stored penile and vaginal swabs, we will conduct proteomic analysis with the supernatant. Protein content will be determined using the Quanti-Pro bicinchoninic acid assay kit (Sigma-Aldrich, MO, USA). The samples will be incubated overnight with trypsin (Promega, WI, USA) and the peptides subjected to metaproteomic analysis using liquid chromatography-tandem mass spectrometry on a Q-Exactive quadrupole-Orbitrap MS (Thermo Fisher Scientific, MA, USA) coupled with a Dionex UltiMate 3000 nano-UPLC system (120 min per sample). Proteins will be identified using a custom UniProt database including human and vaginal microbial entries. Taxonomy will be assigned using UniProt and relative abundance of each taxon will be determined by aggregating the intensity-based absolute quantification (iBAQ) values of all proteins identified for each taxon [41].

#### Documenting relationship break-ups in study couples

If a couple breaks up during Igugu Lethu, each partner will be invited to complete a break-up questionnaire over the phone. The questionnaire will document when the couple broke up if they took up the couples’ health screening visit or tested elsewhere for HIV as an individual/couple between their most recent Igugu Lethu data collection visit and the break-up, and whether or not they feel their participation in Igugu Lethu contributed to the break-up. After completing the break-up questionnaire, follow-up of the couple ends, and study staff will no longer contact them. All participants will be provided with referrals to local counselling/support centres.

#### Process evaluation

The acceptability of the intervention and participation will be evaluated during the study. A sub-sample of participants will be invited by study staff to take part in semi-structured process interviews, with a gender-matched interviewer, at various time points during and after the intervention. Participants will be sampled purposively based on gender, age, engagement with the intervention, and engagement with the general couples’ health screening. Topics explored will include experiences of the intervention and perceptions about attending couples testing. We anticipate recruiting a sample of 20–30 participants, ideally including partners from within the same couple. In addition, all participants will be asked process evaluation questions as part of the 4-month questionnaire. Process interviews or focus groups will also be conducted with up to 5 staff members to explore their experiences of delivering the intervention.

### Outcomes

#### Primary outcome

The primary outcome measure is the proportion of couples that take up couples HIV testing as part of a health screening visit during study follow-up i.e. within 4 months from the date of the first group session, compared to the equivalent proportion in the intervention arm of the Uthando Lwethu study (historical controls).

#### Secondary outcomes of the study

The following secondary outcomes will be measured among couples who participated in the couple’s health screening visit:

- Concordance of HIV test results among couples
- Uptake of blood pressure (BP) measurement as a couple
- Concordance of hypertension diagnosis (defined as > 140/90 mmHg) within couples who take up couple’s BP measurements
- Uptake of sexually transmitted infection (STI) testing as a couple
- Concordance of STI test results within couples who take up couple’s STI testing
- Uptake of random blood glucose (RBG) finger-prick test for diabetes as a couple
- Concordance of diabetes diagnosis (defined as RBG > 11.0 mmol/L) within couples who take up the couple’s RBG testing
- Uptake of height and weight measurement for the calculation of body mass index (BMI) as a couple
- Obesity (BMI ≥ 30 kg/m2) concordance within couples
- Acceptability of self-sampling for STI testing, measured using Likert scales asked as part of the couple’s health screening questionnaire
- DNA yield, and DNA analysis, of the penile swab samples self-collected by men for STI testing, measured using DNA amplification methods
- Penile microbiome functional characteristics of male partners using stored penile swabs, assessed using metaproteomics analyses
- Vaginal microbiome functional characteristics of female partners using stored vaginal swabs, assessed using metaproteomics analyses
- Concordance of results from metaproteomics analyses of penile and vaginal swabs within couples

Additional secondary outcomes are:

o. Acceptability of the intervention and study participation using findings from individual, semi-structured process interviews
p. Whether relationship dynamics are factors in achieving the outcome of couples’ uptake of HIV testing together, using relationship scale variables collected at baseline, week 4, and 4 months

### Statistical considerations

#### Sample size, accrual, follow-up

In the Uthando Lwethu study, 39% of the intervention arm (65/168 couples) had taken up testing together for HIV by 4-months follow-up. A sample size of 180 couples in the Igugu Lethu study provides more than 85% power, at the 5% level, to detect a 40% increase in the proportion testing together, i.e. change from 39 to 55% between the two versions of the intervention, and allow for 5% lost to follow-up or break-up by 4-months.

We aim to accrue the sample of couples over approximately 16 months of active recruitment. Given the continued SARS-CoV-2 pandemic, changes in public health and research-related restrictions may mean a slower rate of recruitment. We will recruit in waves and aim to have approximately 20 couples participate in a round of intervention activities, and will continue to conduct intervention rounds until we achieve our goal of 180 couples. We will follow the couples for a total of 4-months from the date of the first group session in each intervention round.

#### Data analysis

Our primary analysis will compare the proportion of couples who test for HIV in Igugu Lethu with the proportion of couples in the intervention arm of the Uthando Lwethu study who tested for HIV within 4-months of follow-up, unadjusted, using a chi-square test. We will also compare this cohort’s baseline characteristics with the Uthando Lwethu cohort’s baseline characteristics. If there are substantial differences, the comparison will be adjusted for these characteristics using logistic regression.

We will also consider the proportion of couples taking up each of the other non-HIV tests offered as part of the couples health screening. We will calculate descriptive statistics and apply chi-square tests to examine concordance in test results within couples and the secondary outcomes listed a)-i). The acceptability of self-sampling for STI testing by men and women will be examined using responses to Likert scales asked as part of the couple’s health screening questionnaire, separately for men and women, and compared within couples.

The content of the intervention is designed to enhance couples’ levels of communication, intimacy, and other positive relationship characteristics. Therefore, we expect that as couples’ levels of positive relationship characteristics increase, they will be more likely to participate in couples HIV testing. To evaluate the extent to which relationship dynamics are associated with couples’ uptake of HIV testing together, we will describe the change in relationship scores between baseline and 4-weeks, and baseline and 4-months. We will test for associations between these score changes and the HIV couples testing outcome using logistic regression. In addition, the acceptability of the intervention and study participation will be explored through thematic analysis using findings from individual, semi-structured process interviews, and descriptive statistics of responses to the process evaluation questions asked of all participants as part of the 4-month questionnaire.

DNA yield from stored penile swabs will be used to determine whether men’s self-sampling around the coronal sulcus is a feasible method for sample collection, an important finding for future studies. Assuming sufficient DNA for 16S analysis and protein for proteomic analysis from the stored swabs, vaginal and penile microbiome composition will be described. For functional analysis, iBAQ values of proteins with the same Gene Ontology IDs will be aggregated. Basic R functions and the ggplot2 R package will be used for data manipulation, transformation, normalisation and generation of graphics. Data will be further explored using principal component analysis using the mixOmics R package, unsupervised hierarchical clustering using the Complex Heatmap package and FlipPlots. The limma R package will be used to identify differentially abundant taxa, proteins and gene ontologies within couples and between groups defined by age, STI status, and 16S profiles.

#### Ethical considerations

Public involvement in community-based health research in South Africa is well-established and monitored. Community involvement is integral to the development of all research proposals supported by HSRC at the HSRC Sweetwaters site. A community advisory board (CAB) includes representatives of the provisional health and welfare departments and municipal government, and community leaders. The CAB meets quarterly with Sweetwaters senior staff to discusses all study proposals and ongoing projects, community research priorities, and community issues that arise.

The Igugu Lethu study has convened a dedicated community working group (CWG) of 8–10 members including at least one CAB member. The CWG will liaise closely with the study team throughout the study, including through quarterly meetings. The CWG received additional training to facilitate contributions to decisions about study-specific operations, particularly focusing on ensuring feasibility and acceptability in the communities they represent. The study was approved by the HSRC Research Ethics Committee, South Africa (2/19/10/11c), the Faculty of Medicine Research Ethics Committee, University of Southampton, UK (53709), and the Health Research Committee of the KwaZulu-Natal Provincial Department of Health (KZ_202009_041). A data monitoring committee was not required for this low-risk behavioural intervention cohort study.

#### Informed consent

The HSRC Sweetwaters site is experienced in obtaining consent from couples in prior studies [9, 42]. Before a recruiter administers the screening questionnaire over the phone, verbal consent will be recorded. When the call has ended, the audio recording will be uploaded by the recruiter to a password protected, encrypted computer file, labelled with the individual’s study ID, stored separately from the study data.

Written informed consent will be required before conducting the baseline screening, for the couples health visit and for process interviews (invited participants and staff). Each member of the couple will consent individually. If participants cannot provide a signature due to literacy issues, a witness will sign on their behalf. The participant information and consent documents will be available in English and isiZulu. All participants will be given a copy of each participant information and consent document they sign to take home. Separate consent will be sought from couples to audio record their couples counselling sessions for quality assurance and for research purposes, and to audio-record process interviews. As part of the informed consent process for the couples health visit, participants will be asked to consent to their contact details and the outcomes of their health check being shared with the Department of Health (DoH). This is per the site’s memorandum of understanding with the DoH, 14/8/3/1/2179 so that the DoH has a more complete medical history for each participant and can provide uninterrupted care. Results of the health screening visit will only be shared with the study counsellor by the participants themselves. Couples may withdraw from the study at any time.

#### Risks

The possibility exists that participating in the study might lead to tension, conflict and/or possible violence between the partners. The risk for participants primarily stem from 1) discomfort or distress as a result of the personal and sensitive nature of the assessment interviews and intervention sessions or 2) loss of confidentiality. For the optional procedures of HIV and STI-testing, there are the following risks: 1) there are small risks associated with the finger prick necessary for use of the rapid HIV test, 2) risks associated with finding out that oneself or one’s partner is HIV-positive/STI-positive, 3) the possibility that some participants may feel pressure to test for HIV. Finally, there is additional potential for risk should the study team become aware of situations in which HIV/STIs transmission could occur.

Risks to subjects will be minimised by: 1) training of staff in the ethical conduct of research and all confidentiality-protecting procedures 2) training of staff in issues specifically pertaining to couples in this setting (e.g. potential for coercion for women participating, potential for partner violence, possible coercion to test for HIV), 3) close monitoring of any adverse events with appropriate IRB reporting and 4) referral to social workers if a staff member has concerns about a participant’s safety or well-being. One area where confidentiality cannot be guaranteed by the study team is information shared by participants in in group sessions. However, we will directly address the importance of respecting group members’ confidentiality at the start of the session when study facilitators and participants agree ground rules.

Additional measures are needed to protect each participant’s confidentiality from their partner. In previous couples-focused research, we have developed procedures that include conducting individual data collection interviews by a gender-matched interviewer in a private room, ensuring that each member of couple does not have contact with the interviewer who administered the baseline and follow-up surveys to their partner. During the informed consent process at the baseline screening, we will discuss with participants the possibility that their partner might ask about their answers to certain questions or issues they discussed. Interviewers will be trained to discuss with participants ways of coping with this situation if the participant they are consenting expresses concern or distress about this matter either during consent, the administration of a questionnaire, or after a questionnaire is completed. Interviewers and intervention staff will also be trained in the identification of, and proper response to, issues of coercion or abuse, and will be know how to refer participants for domestic violence assistance. A list of community-based resources for couples is regularly updated, and will be offered to each couple at all visits.

#### Risk of HIV disclosure

All staff will be familiar with Confidentiality: Protecting and providing information (Booklet 5) from the Health Professions Council of South Africa’s guidelines for HIV disclosure [43]. The South African Medical Association recommends that health care workers do not disclose the HIV status of an individual to third parties (including family, sexual partners, and employers) unless the patient has provided informed consent. At present, there is no law in South Africa forcing people to disclose their HIV status to their partner. Should the situation arise where it is established that one partner has not disclosed their HIV-positive status to their partner and they are not on antiretroviral therapy, study couples counsellors will offer support to the participant to disclose to their partner and refer for further support if needed. The study team would also actively facilitate linkage to care to initiate ART, counsel to abstain from sex or consistently use condoms.

#### Data management

The HSRC Sweetwaters team has successfully used mobile phones for data collection on several large scale evaluations and projects, including the Uthando Lwethu Study. Mobile phones will be used to collect all operational and research data through the mobile phone application by Mobenzi Researcher (Durban, South Africa). Data are temporarily stored in a non-readable encrypted file on the handset until an area with network coverage is reached and the data are uploaded to a secured server at the HSRC Sweetwaters site and removed from the mobile phone. Data security is enhanced by authentication protocols.

Participants’ names will not be associated with any physical or digital data collected or managed by the study, only research identification numbers will be used. Any tracing or other contact information, including signed consent forms will be stored separately from survey data and all records will be stored in locked file cabinets in study offices at HSRC. The files linking research identification numbers and names will be stored in a separate locked file cabinet, and a computer file only accessible by the Project Director, PI and Co-Is; and all computers will be password protected.

#### Adverse event reporting

All safety-related risks will be monitored routinely during data collection visits and intervention sessions. Any incidents involving subject safety will be reported by the PI to both institutional ethics committees (Human Sciences Research Council and University of Southampton) within 10 working days. The report will include information on 1) all serious adverse events associated with study procedures and/or 2) any events or problems involving the conduct of the study or patient participation, including problems with the recruitment or consent processes.

#### Impact of the COVID-19 pandemic

The COVID-19 pandemic has been a challenge for the Igugu Lethu study. Recruitment was scheduled to start April 1st, 2020, when South Africa declared a State of Disaster on March 15th, 2020. Initial restrictions on international travel, public gatherings and daily curfews were followed by a strict lock-down from 26th March 2020, resulting in study recruitment beginning in December 2020. With the various and continued COVID-19 pandemic restrictions and impacts, the study team have needed to repeatedly revise operational management to protect staff and participants. When all public gatherings were banned, the study team tried unsuccessfully to organise online group sessions with couples. Only 9% of the couples in our study report that both partners own a smartphone. Alternative arrangements such as lending smartphones to participants or using audio conferencing equipment to gather couples together were not feasible in the study setting. When government restrictions allow, we have undertaken community meetings and promoted the study more widely within the community. The study was further challenged when, due to the impact of the COVID-19 pandemic, Mobenzi, the South African software company being used by the study discontinued its support for mobile data collection at the end of December 2021. After considerable efforts by the study team, data collection was transitioned to REDCap (Richmond, VA; Vanderbilt University).

Given the impacts of the COVID-19 pandemic, we have reflected on the appropriateness of using historical controls to evaluate the effectiveness of the optimised intervention. The experience of lockdown may have changed levels and patterns of sexual activity, particularly of couples who are not living together. This in turn may have changed couples’ perceptions of their HIV risk and their need to know their current HIV status. In KZN province, at the start of the pandemic, use of HIV testing services declined but gradually improved as restrictions eased towards pre-lockdown levels by August 2020 [44]. Specific data regarding provision and uptake of couples HIV testing in KZN during this period are not available, however it is expected that these will have followed a similar pattern, and not be any higher than pre-pandemic low levels [9]. Key characteristics related to HIV risk are measured in the Igugu Lethu study. In analyses, the Igugu Lethu cohort’s baseline characteristics will be compared with the baseline characteristics of the historical controls from the Uthando Lethu study. Therefore, we can adjust analyses for any important differences.

## Discussion

By strengthening communication and functioning within the relationship, both the Igugu Lethu and Uthando Lwethu studies aim to transform the motivation of individual partners from a focus on their own health to shared health as a couple [12]. The Igugu Lethu study aims to achieve a 40% higher proportion of couples testing for HIV together than the Uthando Lwethu study. The optimised intervention has four new aspects: (i) community members speak publicly about their HIV-testing history, HIV status, and how testing impacted their relationships as a starting point for group discussion around couples testing; (ii) couples are split into separate single-gender groups earlier than in the Uthando Lwethu study. This provides more time in ‘safe’ spaces to open up and discuss health and relationship issues, prepare themselves for the counselling sessions, thereby priming the couple to gain greater benefit from the counselling process and feel more ready to test; (iii) greater attention is given to assisting couples to understand the consequences of testing, change outcome expectancies, and manage negative emotions around HIV testing. In the counselling sessions, couples will practise their communication and problem-solving skills to discuss HIV-specific issues in their relationship. We anticipate that these relationship strengthening efforts will also have long term benefits for the couple, for example, reducing the risk of IPV; (iv) participants’ knowledge of adult health is broadened to include sexually transmitted infections and the interrelationships between weight, BMI and non-communicable diseases such as diabetes and hypertension in the first group session. In addition to standard CHTC, participants are offered an opportunity to test for other health conditions including STIs.

Igugu Lethu study findings regarding uptake levels of other health screening by couples that have chosen to take up CHTC, can inform differentiated HIV testing strategies [45], differentiated HIV service delivery [46] and taking such interventions to scale [47]. Offering health screening outside of the clinic setting may also mean that men who have been previously hard-to-reach for HIV testing and care could be reached as male partners [16, 48]. The acceptability and feasibility of asking participants, in the context of a couple, to privately self-sample for STI testing will also be important for future research and practice. Providing STI results to couples is also an opportunity for treating asymptomatic cases and promoting reproductive health, facilitating partner treatment and reducing onward STI transmission.

The main hypothesis of the Igugu Lethu study is that our optimised couples-focused behavioural intervention and offering a broader health screening alongside couples HIV testing will increase demand for CHTC. CHTC has already been shown to be cost-effective and to contribute to preventing new HIV infections [49], an outcome that is highly desirable in populations with high HIV prevalence.

## Supplementary Information

**Additional file 1.**

## Data Availability

The anonymised datasets generated during the study will be available on the University of Southampton data repository, eprints.soton.ac.uk. In accordance with the University of Southampton’s data policy, the data will be archived from a minimum of 10 years after publication or last access, whichever is longer. DOIs will be issued for the dataset and data subsets in line with the University’s DOI policy.

## Abbreviations

HIV: Human Immunodeficiency Virus
CHTC: Couples HIV testing and counselling
UL: Uthando Lwethu
KZN: KwaZulu-Natal
IPV: Intimate partner violence
HSRC: Human Sciences Research Council
CAB: Community advisory board
CWG: Community working group
NCD: Non-communicable disease
BMI: Body mass index
STI: Sexually transmitted infection
WHO: World Health Organisation
DOH: Department of Health
RBG: Random blood glucose
BP: Blood pressure
SBP: Systolic blood pressure
DBP: Diastolic blood pressure
DNA: Deoxyribonucleic acid
TPPA: *Treponema pallidum* particle agglutination
iBAQ: Intensity-based absolute quantification
