# Supplementary material for: Protocol: evaluation of an optimised couples-focused intervention to increase testing for HIV in rural KwaZulu-Natal, South Africa, the Igugu Lethu (‘Our treasure’) cohort study
Source: BMC Public Health. 2022 Aug 19;22:1577. doi: 10.1186/s12889-022-13894-3 (PMC9389496; doi:10.1186/s12889-022-13894-3)
Supplement: Supplementary file 1 — Additional file 1. [file 12889_2022_13894_MOESM1_ESM.docx]

# INFORMATION SHEET AND CONSENT FORM

**Igugu Lethu Study- A Couples-focused Intervention to Increase Testing for Health including HIV and Sexually Transmitted Infections (STIs) Among Heterosexual Couples in Vulindlela, South Africa**

**WHO WE ARE**

Hello, my name is……..………………………...... I am working for the Human Sciences Research Council (HSRC) at the Sweetwaters office.

**WHAT ARE WE DOING?**

We are conducting research to improve the couples counselling intervention that we used in the Uthando Lwethu study and to conduct a new study to test the improved intervention success in promoting couples HIV testing and counselling and couples health in general. You are being invited to participate in this study with your partner. We hope to help you and your partner look after your health, and to learn how participating in a couples-focused study might help your relationship and communication.

This is a joint project between the Human Sciences Research Council and the University of Southampton, UK.

**WHO CAN TAKE PART IN THE STUDY?**

A questionnaire will be used by the study staff member to determine whether you are eligible for this study. You will be asked questions about your relationship with your partner (including how you and your partner communicate and your sexual relationship), your sexual behavior, HIV testing history and health status, and any history of domestic violence. Your answers will be entered on a mobile phone and stored securely. The information we collect will be sent to our authorized study staff only and no one else. You will be interviewed by yourself, separately from your partner. Your responses and those of your partner will be confidential and we shall not disclose your responses to your partner, nor your partner’s responses to you.

During screening, hand sanitizer and masks will be provided for you and staff, and appropriate social distancing and ventilation will be maintained, to ensure a COVID safe environment. Universal wipes will be used to clean fingerprinting scanner, pens and all other surfaces before you use them.

The questionnaire will take approximately sixty minutes. If you are eligible you will be enrolled in the study.

Not all couples will be able to take part in the study and if this is the case for you, the research staff will explain why.

**WHAT WILL IT MEAN TO BE ENROLED IN THIS STUDY?**

There are two types of visits in this study. There are visits where you will receive counselling and support in looking after your health and relationship, and there are data collection interviews.

Counselling and support visits:

Visit 1: You will participate in a group session with your partner. This session will take approximately ½ day and will focus on couples health. For the second half of the session you will be split into male and female groups to talk about topics such as HIV and family planning. This group session will be with up to 20 other couples.

Visit 2: Approximately a week after the first group session, you and your partner will participate in a second group session in male groups and female groups with about 10 other participants. Group leaders will encourage discussions about relationship issues, such as commitment and trust. Training will also be provided about how to communicate about issues such as sex and HIV. This workshop will take approximately 1/2 day.

Visits 3-6: You and your partner will participate in up to four couples’ counselling sessions with a trained couples’ counsellor. There will be specific topics for each session, and you will be provided with some skills training, and an opportunity to discuss your health-related relationship goals with the counsellor. Each session will be approximately 90 minutes to two hours, and will be scheduled at your convenience.

For all of these visits (1-6), hand sanitizer and masks will be provided for you, your partner and staff, and appropriate social distancing and ventilation will be maintained, to ensure a COVID safe environment. Universal wipes will be used to clean fingerprinting scanner, pens and all other surfaces before you use them.

Data collection interviews:

These will be conducted by phone for a maximum of 60 minutes, 4 weeks and 4 months after you start the study. These interviews will cover topics such as HIV/AIDS, and your relationship with your partner. You will be interviewed by yourself, separately from your partner. Your responses and those of your partner will be confidential and we shall not disclose your responses to your partner, nor your partner’s responses to you.

These interviews will be scheduled at your convenience. After 4 months the study will end.

During the study you will be offered the opportunity to take up a couples health screening visit at any time **as a couple.**

**DO I HAVE TO TAKE PART?**

Please understand that **your participation is voluntary** and you are not being forced to take part in this study. The choice of whether to participate or not, is yours alone. If you choose not to take part, you will not be affected in any way whatsoever. If you agree to participate, you may stop participating in the research at any time and tell me that you don’t want to go continue. If you do this, there will be no penalties and you will not be prejudiced in any way.

If you do want to take part, after you have had all your questions answered and feel you understand what you will have to do, you will be asked to sign, or put your thumbprint on a consent form. If you cannot read then we recommend that you have a witness present for the discussion and to see your thumbprint. You are not obliged to have someone present. This confirms that you are willing to take part in this study. You will then be given a number which is unique to you and which will help us to ensure that all your answers to our questions remain private. We will also ask if you are happy for us to scan your fingerprint to help us re-identify you at later visits. Your fingerprint scan will be stored on a computer in such a way that it cannot be linked to any other data.

**CONFIDENTIALITY**

All **identifying information** will be kept in a locked file cabinet, will not be available to others and will be kept confidential to the extent possible by law. The records from your participation may be reviewed by people responsible for making sure that research is done properly, including members of the ethics committee at the Human Sciences Research Council and the University of Southampton, (all of these people are required to keep your identity confidential). Otherwise, records that identify you will be available only to people working on the study, unless you give permission for other people to see the records. Your personal sensitive information will not be disclosed.

**Your answers and any information put into the mobile phone** will be stored electronically in a secure environment and used for research or academic purposes now or at a later date in ways that will not reveal who you are. Any information about you will be identified only by a participant study number. The link between your name and participant study number will be kept in a secure location at the Sweetwaters office only. We will not discuss any information about you. Any publication of this study will not use your name or identify you personally.

When participating in group sessions, your confidentiality cannot be guaranteed, but we will make every effort to protect your confidentiality if you participate. Please do not share any personal or sensitive information in the group sessions.

**Please be aware that you will not be told any information that your partner says in his or her interview.** This includes any information your partner might say about his or her sexual health, including HIV status, even if we think you do not know this information. Likewise, no information you say in your interview will be told to your partner, even if we think your partner might not know this information.

If you are HIV-positive, not currently taking treatment for HIV, fail to disclose your HIV-positive status to and engage in unprotected sex with your HIV-negative primary partner, you will be referred to the study counsellor for sexual risk reduction counselling. He/she will help you develop a plan of action for disclosing to your HIV negative primary partner. If necessary, follow-up will be provided or you will be given the names of referral resources in the community that could provide you with additional help in disclosing your HIV infection to your primary partner. However, please understand that there are limits to confidentiality, such that the investigators may be required by law to take steps, including reporting to the authorities, in the event that you may be a threat to your own safety or that of others.

**FUNDING SOURCE**

The study is funded by the NIHR (National Institute of Health Research).

**RISKS/DISCOMFORTS**

Some of the topics in the questionnaire interview are personal and you may feel embarrassed or uncomfortable. However, you may skip any question at any time. You may stop the interview at any time. You may decline to participate in any part of the group discussions, and you may leave the group discussions at any time. If you become distressed or uncomfortable during a counselling session, you may stop the session at any time.

It is possible that your partner may ask you about your responses to certain questions or issues that come up in the interview. This may cause some discomfort or distress in your relationship with your partner.

Should you feel any discomfort or distress, you can consult one of the study counsellors. In addition, all participants will receive a list of community-based resources, including mental health counselling, general health services, and other issues.

It is important that you know that you do not have to be in this study if you do not want to join. Even if you decide to participate, you can withdraw at any time. If you withdraw, you will not be prevented from accessing any services or help that you need. You have the right to decline any of the study procedures at any time. If you do withdraw, we will attempt to contact you to check if you are happy for us to use the information you have already provided.

**BENEFITS**

You will receive counselling that may improve your communication with your partner and have other positive impacts on your relationship. You may also learn some information about general health issues and HIV and about resources in your community.

In addition, the information gained from the study may provide information that is very important for community organizations, researchers at HSRC, and health workers in this area to provide better services for couples in Vulindlela.

**COSTS**

There will be no cost to you as a result of taking part in this study as we will be traveling to meet you in a location that is convenient for you for data collection interviews and providing transport for you to all the counselling and support visits.

# PAYMENT

In return for your time, you will be reimbursed ZAR 80 for each of the data collection interviews i.e. you will receive ZAR 80 for the baseline assessment, ZAR 80 for the 4-week assessment, and ZAR 80 for the 4-month assessment. These reimbursements will be by universal cell-phone voucher, sent electronically to your phone to avoid between person contact.

**WHO TO CONTACT IF YOU HAVE BEEN HARMED OR HAVE ANY CONCERNS**

If you any complaints about ethical aspects of the research or feel that you have been harmed in any way by participating in this study, please call the HSRC’s toll free ethics hotline 0800 212 123 (when phoned from a landline from within South Africa) or the research ethics committee (REC) Administrator, Khutso Sithole at the Human Sciences Research Council on 012 302 2009. You may also contact the administrator on the following email address: research.ethics@hsrc.ac.za.

If you have concerns or questions about the research you may call the project leader, [Nkosinathi](mailto:Nkosinathi) Ngcobo (082 397 1464 and email:NNgcobo@hsrc.ac.za).

You will receive a copy of this form today.

**Data Protection Privacy Notice**

The University of Southampton takes your participation in this study very seriously and wants to make sure the researchers are held to the highest standards of research integrity. The University of Southampton has to ensure that any personally-identifiable information we collect is in the public interest. This means that when you agree to take part in this study, your personal data can only be used by the researchers in the ways needed, and for the purposes specified, to conduct and complete the research project. Under the data protection law, ‘Personal data’ means any information that relates to and is capable of identifying a living individual.

“Personal data” (or “Personal information”) is also protected by the Protection of Personal Information Act (PoPI Act) which was signed into law in South Africa by President Zuma in 2013. The PoPI Act aims to ensure that the right to privacy is protected against the unlawful collection, retention, dissemination and use of personal information. You can find more information about the PoPI Act on its website <https://www.saica.co.za/Technical/LegalandGovernance/Legislation/ProtectionofPersonalInformationAct/tabid/3335/language/en-ZA/Default.aspx> [Accessed 9/5/2019].

This Participant Information Sheet tells you what data will be collected for this project and whether this includes any personal data. Please ask the research team if you have any questions or are unclear what data are being collected about you.

Any personal data we collect in this study will be used only for the purposes of carrying out our research and will be handled according to the University of Southampton’s and HSRC’s policies in line with data protection law. If any personal data are used from which you can be identified directly, it will not be disclosed to anyone else without your consent unless the University of Southampton or HSRC are required by law to disclose it.

Data protection law requires us to have a valid legal reason (‘lawful basis’) to process and use your Personal data. The lawful basis for processing personal information in this research study is for the performance of a task carried out in the public interest. Personal data collected for research will not be used for any other purpose.

For the purposes of data protection law, the University of Southampton is the ‘Data Controller’ for this study, which means the institution is responsible for thinking about why we want to collect this information from you, how it is collected from you and making sure your data is carefully handled. The University of Southampton will keep identifiable information about you for 15 years after the study has finished after which time any link between you and your information will be removed. The HSRC will also keep identifiable information about you from this study for 15 years after the study has finished.

To protect your rights, we will use the minimum personal data necessary to achieve our research study objectives. Your data protection rights may be limited, however, in order for the research output to be reliable and accurate. The University of Southampton will not do anything with your personal data that you would not reasonably expect.

# CONSENT FORM - INDIVIDUALS

**Igugu Lethu Study - A Couples-focused Intervention to Increase Testing for Health including HIV and Sexually Transmitted Infections (STIs) Among Heterosexual Couples in Vulindlela, South Africa**

***Please initial the box(es) if you agree with the statement(s):***

| I have read and understood the information sheet, version 1.3, dated 03/11/2020, and have had the opportunity to ask questions about the study. |  |
| --- | --- |
| I agree to take part in the Igugu Lethu study and agree for my data to be used for the purpose of this study. |  |
| I understand that I am participating freely and without being forced in any way to do so. |  |
| I understand that I can stop participating at any point should I not want to continue and that this decision will not in any way affect me negatively. I understand that if I withdraw the study team will attempt to contact me to check if I am happy for the information I have already provided to be used. |  |
| I understand that this is a research project whose purpose may not benefit me personally in the immediate or short term. I understand that my participation will remain confidential. |  |
| I understand that I will not be told any information that my partner says in his or her interview |  |
| I understand that the information that I provide will be stored electronically and will be used for research purposes now or at a later stage |  |

The following are optional to agree or disagree, please initial:

|  | Yes | No |
| --- | --- | --- |
| I agree to have my fingerprint scanned for this study and I understand that my fingerprint will be used for the purpose of re-identifying me at later study visits. |  |  |
| Do you agree to be contacted by phone in order to follow-up with you if you miss an appointment? |  |  |
| Do you agree to be visited at home if you miss a study visit? |  |  |

**[Note: Your answers will not affect your participation in any way]**

Name of participant (print name)…………………………………………………

Signature of participant…………………………………………………….

Date……………………………………………………………………….

Name of study staff member (print name)……………………………………

Signature of study staff member ……………………………………………

Date…………………………………

***The following only applies if the participant has a witness***

Name of witness (print name)… ………………………………… ………

Signature of witness …………………………………………………….

Date…………………………………………………………………………..

***Give a copy to participant***
